# An introduction to the journal review and editorial process

**DOI:** 10.1016/j.jvscit.2025.101745

**Published:** 2025-01-31

**Authors:** Ben Li, Matthew R. Smeds

**Affiliations:** a Division of Vascular Surgery, Department of Surgery, University of Toronto, Toronto, Ontario, Canada; b Division of Vascular and Endovascular Surgery, Department of Surgery, Saint Louis University, St. Louis, MO

Preparing, revising, and finalizing a manuscript for publication can be a challenging process, particularly for trainees and early-career researchers.^1^ Understanding the journal review and editorial process can provide authors with insights into how to effectively write, format, and submit initial and revised versions of their manuscripts to increase the potential for publication.^1^ In this article, we describe key aspects of the journal review and editorial process from submission to publication, with specific examples from the *Journal of Vascular Surgery Cases, Innovations and Techniques* (JVSCIT) (Fig).

**Fig:**
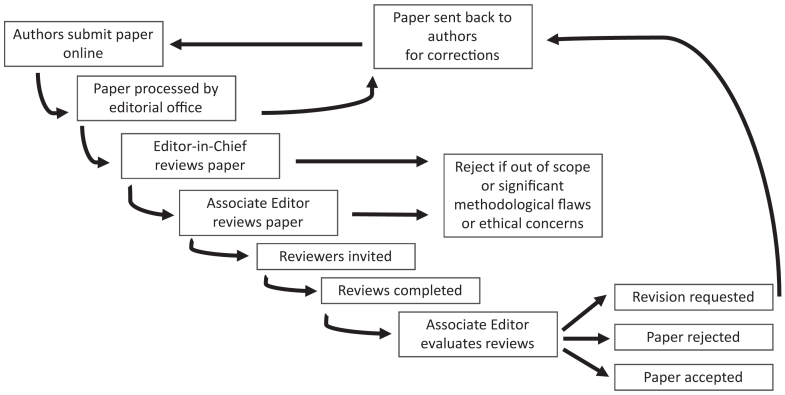
Summary of the journal review and editorial process at the *Journal of Vascular Surgery Cases, Innovations and Techniques*.

## Step 1: Editorial office checks manuscript against journal requirements

Authors should carefully read the journal's instructions for authors before submitting their manuscript.^2^ This comprehensive document generally outlines the aims and scope of the journal, article types accepted, formatting and content requirements, and publication fees, among other important information.^2^ All submissions to JVSCIT are checked thoroughly by the editorial office to ensure that they meet journal requirements.^3^ Manuscripts with missing information or inappropriate structure or style are sent back to the authors for revision, delaying the editorial process.^3^ Additionally, most journals, including JVSCIT, will put the manuscript through plagiarism detection software to check for similarities with existing articles.^4^ Articles with high similarity scores are investigated for duplication of work or appropriation of content from other articles, with clarification requested from the authors if unable to be adjudicated. Therefore, it is important that authors submit work that is original and referenced appropriately.^3^ With the popularization of generative artificial intelligence (AI) tools, it is important that authors follow guidance provided by journals regarding the appropriate use of generative AI in scientific writing.^5^ JVSCIT states that authors must disclose the use of generative AI and AI-assisted technologies in the writing process, including the name of the tool or service and its purpose, but not list AI tools in the author list. Authors must take full responsibility for the content of their manuscript, even that which has been generated by these instruments.^3^ By taking steps to follow journal-specific guidelines, authors help to maintain ethics in publishing and will streamline the editorial handling process.^6^

## Step 2: Editors perform an initial review of the manuscript

Once the manuscript is deemed by the editorial office to have met journal requirements, it is sent to a senior editor for an initial review.^3^ At JVSCIT, the editor-in-chief performs an initial review of all submissions.^3^ Some manuscripts may be rejected at this point, primarily because they are out of the journal's scope or have significant methodological flaws or ethical concerns.^3^ If these issues are absent, the manuscript is assigned to an associate editor for handling.^3^ Once the associate editor has reviewed the manuscript and believes that it has potential for publication, it is sent for peer review.^3^

## Step 3: Peer review of the manuscript

Peer review is fundamental to the editorial process, whereby the handling editor sends the article to experts in the field related to the manuscript for their assessment.^7^ These experts are chosen by the handling editor, generally based on existing journal reviewers and/or editorial board members who have demonstrated expertise in the field, authors who have published similar articles on the topic, and/or recommendations of the authors, who can specify preferred or opposed reviewers based on expertise and/or conflicts of interest.^3^ At JVSCIT, there is a pool of potential reviewers. Although we are always looking for additional engaged and enthusiastic reviewers, authors are asked only to provide a list of opposed reviewers.^3^ These reviewers are people with a potential conflict of interest or bias who the authors feel may be selected to review their manuscript. Reviewers are given specific instructions on how to assess the manuscript, write a review, and provide a recommended course of action.^3^ These recommendations are generally to accept, reject, or revise (major or minor) the manuscript, which briefly means the following:

1. Accept – the manuscript can be accepted without changes.
2. Reject – there are fundamental flaws in the manuscript and the reviewer does not believe these can be adequately addressed with revisions.
3. Major revision – there is some potential to publish the manuscript given significant changes to the design, methodology, reporting, and/or other scientific aspects of the study. Acceptance is not guaranteed after revision.
4. Minor revision – there is good potential to publish the manuscript given minor changes to the structure, wording, and/or other formatting (or minor scientific) aspects of the manuscript. At JVSCIT, this is considered a provisional acceptance, meaning that, if the reviewers' minor concerns are addressed adequately, most papers will be accepted.

JVSCIT has historically used a single-blinded review process, whereby the authors do not know the identities of the reviewers, but the reviewers know the identities of the authors and their institutions.^8^ However, we have recently begun a trial process of double-anonymous peer review, whereby the identities of both the reviewers and authors are concealed from each other.^8^ Through a double-blinded peer review process, the potential for conscious or unconscious bias by the reviewers based on the identities or affiliated institutions of the authors may be reduced, allowing for a more merit-based assessment of the scientific value of the manuscript.^3^ At JVSCIT, the goal is for three reviewers to assess every manuscript, with additional reviewers as needed, usually in cases of discrepancies in evaluations or inadequate reviewer comments.^3^ Reviewers provide an assessment of the paper with recommendations regarding acceptance as well as comments to the authors that may help them to improve the quality of the manuscript. An open access how-to guide for JVSCIT reviewers outlines key considerations for high-quality reviews including honest and constructive feedback that is clear, specific, concise, and respectful.^9^ Once sufficient reviews are received, the handling editor makes a decision on the manuscript (accept, reject, or major/minor revisions) and sends a decision letter to the authors, including comments from the reviewers and editor(s) if applicable.^3^ If a revision decision is sent, the authors are given a time window (generally 4 weeks at JVSCIT) to respond to the reviewers' and editor's comments and revise their manuscript.^3^

## Step 4: Evaluation of the revised manuscript

Appropriate revision of the manuscript based on reviewer and editor feedback is critical to the success of the manuscript.^10^ At JVSCIT, authors are asked to provide a point-by-point response to each reviewer/editor comment in a structured reviewer response form.^3^ Particularly important comments are marked with an asterisk and should result in an appropriate change in the manuscript.^3^ All changes should be documented clearly in both the reviewer response form and a redline manuscript, and all reviewer questions should be addressed.^3^ Once a revised manuscript is submitted, it is reviewed by the handling editor.^3^ If significant revisions have been made, the manuscript is generally sent back to the original reviewers, who will re-evaluate the revised manuscript and provide an updated recommendation based on whether the comments have been addressed sufficiently.^3^ If minor revisions have been made, and the handling editor does not require re-review by the original reviewers, a final decision can be made.^3^ A frequent cause of nonacceptance with request for more revisions after the initial decision is inadequate response to reviewer questions either by not answering a question or answering a question with a good response but not incorporating this response in the revised manuscript or providing a reason why this should not be included in the manuscript. Authors can minimize revision requests by fully adjudicating all reviewer comments.

## Step 5: Final editorial decision

The decision made by the handling editor generally depends on the recommendations of the reviewers and an assessment of whether the manuscript is novel, methodologically sound, and has potential for important clinical/research impact.^3^ At JVSCIT, once the handling editor decides to accept or reject a manuscript, the decision is sent to the editor-in-chief, who will evaluate the entire review process and send a final decision letter to the authors.^3^ If a manuscript is rejected, a reason is provided to the authors along with relevant reviewer/editor comments and a potential option to transfer the manuscript to a partner journal.^3^ If a manuscript is accepted, it will go into production, whereby a proof is prepared and reviewed by the authors before publication.^3^

## Conclusions

The journal review and editorial process is detailed and systematic to ensure that the highest quality work is published. We have highlighted key steps in this process and provided recommendations for authors to increase the potential for their manuscripts to be published and decrease the handling time of their manuscripts. JVSCIT is committed to publishing high quality work from researchers at all levels, including trainees, and we hope that this article provides some insights into how a manuscript is evaluated for publication. The editorial team at JVSCIT provides support to authors at all stages of the editorial process, from presubmission inquiries through to postpublication promotional activities and can be contacted at jvascsurg@vascularsociety.org.

## Funding

None.

## Disclosures

None.
